# Attentional Bias for Imperfect Pictures in Perfectionism: An Eye-Movement Study

**DOI:** 10.3389/fpsyg.2020.566482

**Published:** 2020-10-02

**Authors:** Juan Li, Xiping Liu, Bin Yu, Weihai Tang, Xinchun Liu

**Affiliations:** ^1^Faculty of Psychology, Tianjin Normal University, Tianjin, China; ^2^Department of Psychiatry and Psychology, Tianjin Medical University, Tianjin, China; ^3^Institute of Applied Psychology, Tianjin University, Tianjin, China

**Keywords:** attentional bias, imperfect pictures, perfectionism, eye movements, visual attention task

## Abstract

**Background:**

Although dot-probe paradigms have been widely used in previous studies to investigate the attentional bias of perfectionists, the exact characteristics of this bias are still unclear.

**Methods:**

In this study, eye-tracking technology was used to compare the attentional patterns of high perfectionists (HP) and low perfectionists (LP). The HP and LP groups (*n* = 39 vs 34) completed a visual attention task in which they observed perfect vs imperfect picture pairs, during which their eye movements (EMs) were recorded automatically using an EM tracking system.

**Results:**

Both the HP and LP groups showed an overall attentional bias toward imperfect pictures, as indicated by the criteria of initial visual attention orientation and attentional maintenance. There were no significant differences between the HP and LP groups during the early and middle phases of attention: both groups exhibited a longer total fixation duration on imperfect pictures than on perfect pictures. However, during the late phase of attention, the participants in the HP group diverted their attention away from the imperfect pictures and began to pay more attention to the perfect pictures. By contrast, the participants in the LP group consistently exhibited longer fixation times for imperfect pictures than the HP group during the entire duration of the stimulus.

**Conclusion:**

These findings indicate that the participants in the HP group tended to avoid imperfect stimuli during the late phase of attention; this may indicate that avoidance plays an important role in maintaining perfectionism. This study also shows that eye-tracking is a useful methodology for measuring the attentional biases of perfectionists.

## Introduction

Perfectionism is marked by a tendency to hold oneself to impossibly high standards and to engage in a high level of self-criticism (Frost et al., 1990). Numerous studies have found that perfectionism is a transdiagnostic process of multiple psychological disorders, and it has been affirmed as an important predisposing and perpetuating factor for such conditions as eating disorders (Boone et al., 2012; Johnston et al., 2018), anxiety (Gnilka et al., 2012; Mitchell et al., 2013), depression (Dry et al., 2015; Mehr and Adams, 2016), and self-harm (O’Connor et al., 2010).

### Theoretical Models of Perfectionism

The cognitive–behavioral model of perfectionism has been developed to account for the maintenance of perfectionism in psychological disorders (Shafran et al., 2002, 2010). The model indicates that a number of cognitive biases underlie perfectionism. One such bias is an attentional bias that causes perfectionists to pay more attention to imperfect stimuli. Such attentional bias is characteristic of clinical perfectionism and involves an individual focusing on a mistake or error they have made even if it is comparatively unimportant (e.g., “One punctuation mistake in a document is evidence that I am not good enough at work”) (Egan et al., 2016).

### Attentional Biases and Perfectionism

Although attentional bias is regarded as an important maintenance factor in the cognitive–behavioral model of perfectionism, the specific details of the attentional process and the precise nature of the attentional biases remain unclear. To date, studies of attentional bias in perfectionism are very sparse, and what work has been done has typically used words and pictures as stimuli to examine attentional processes. For example, Kobori and Tanno (2012) investigated the differences between high perfectionists (HP) and low perfectionists (LP) on an emotional Stroop task that required the participants to color name failure words (e.g., mistake, unsuccess, and error) and neutral words (e.g., newspaper, map, and furniture). Their results suggested that HP take longer to respond to failure words than do LP. Although this finding supports the notion that HP have an attentional bias to negative perfectionism-relevant information, the emotional Stroop task has been questioned as an effective measure of attentional bias (Bar-Haim et al., 2007). Howell et al. (2016) used an attentional probe task to compare the attentional bias of HP and LP as they responded to stimulus words that differed in emotional valence (positive vs negative) and perfectionism relevance (perfectionism-relevant vs irrelevant). The results demonstrated that HP displayed greater attentional preference to negative than to positive information, but only for perfectionism-relevant stimuli. Tonta et al. (2019) further discriminated attentional bias into two subprocesses of attentional engagement and disengagement. They used a modified dot-probe task to evaluate the ways in which perfectionistic concerns and strivings are relevant to attentional engagement and disengagement. Their findings demonstrated that perfectionistic concerns were associated with a disengagement bias for negative stimuli. Although engagement and disengagement bias were calculated by comparing relative latencies to respond to target probes appearing in either the same or opposite locus of the emotional information, they used response latency tasks to indirectly assess attentional bias, and these could not effectively differentiate the different components of attention (Yiend and Mathews, 2005). The results should, therefore, be interpreted with caution.

In addition to using words as stimulus material, pictorial stimuli (e.g., perfect pictures vs imperfect pictures) have also been used in studies. Fei et al. (2011) proposed that perfectionists have imperfection anxiety when they process damaged, unfinished, or untidy stimuli. They compared the difference in attentional preference between HP and LP individuals to perfect and imperfect figures. They found that HP individuals showed attentional bias to an imperfect graph, while LP individuals showed attentional avoidance to an imperfect graph. Based on Fei and his colleagues’ study, Ren and Liu (2013) used pictures of daily life as experimental materials on the ground that they should have higher ecological validity. They further divided LP into positive perfectionists and non-perfectionists. They found that positive perfectionists showed avoidance of imperfect pictures, negative perfectionists showed attentional preference for imperfect pictures, and non-perfectionists did not show any significant attentional bias. The different results between these two studies may be the product of the various grouping criteria used for screening the subjects. Although previous studies have suggested that the perfectionism-related attentional biases posited by the cognitive–behavioral model of perfectionism do indeed exist, the dot-probe tasks used to investigate such biases mainly focused on a “snapshot” picture of attention at one point in time. They were, therefore, unable to capture the time course of attentional processes during exposure to stimuli (Armstrong and Olatunji, 2012).

Attention is a complex cognitive process consisting of initial orientation, engagement, maintenance, disengagement, re-engagement, and avoidance (Fashler and Katz, 2014). Moreover, different components of attention may play different roles at specific stages of attention course. Eye-tracking technology records continuous visual attention (Yang et al., 2012, 2013; Vervoort et al., 2013; Todd et al., 2016) and is thus an excellent tool for studying visual attention at different phases during the presentation of a stimulus. Surprisingly, there has yet been no study using eye-tracking technology to investigate the attentional bias of perfectionists.

### The Current Study

To the best of our knowledge, the present study is the first to empirically test the link between attentional bias and perfectionism using eye-tracking methodology via the investigation of attentional differences between HP and LP groups, thus allowing assessment of visual fixation patterns across relatively long stimulus exposure periods. Based on the cognitive–behavioral model of clinical perfectionism (Shafran et al., 2002), we hypothesized that the HP group would (1) show vigilance toward imperfect pictures in the initial orienting phase, (2) display greater attentional maintenance to imperfect pictures, and (3) show an attentional bias toward imperfect pictures at different stages of visual attentional processing.

## Methods

### Participants

A total of 1205 participants were recruited and asked to complete an online version of the Chinese version of the FMPS (CFMPS) as a prescreening survey, along with several additional screening measures (e.g., SAS and SDS). Individuals with high or low perfectionism screening scores (i.e., ≤45 or ≥78) were selected for recruitment. These cutoffs were determined by calculating the mean and standard deviation of the participants’ total scores on the four negative dimensions of the CFMPS. Participants whose scores were less than or more than one standard deviation from the mean were contacted and invited to participate in the study. Seventy-eight participants were tested approximately 2 months after screening. When they completed the CFMPS a second time, the participants were classified as HP or LP according to the criterion of whether their total scores on the four negative dimensions were below or above the sample median. (The median score for the initial pool of 1205 college students was 61.5). However, four participants were unable to complete the study due to difficulties with calibration of the eye-tracker, and one additional participant was dismissed due to use of psychiatric medication. Thus, seventy-three participants were included in the final analysis. All of these participants had normal or corrected-to-normal vision and reported no past and present neurological disease or psychiatric illness. There were 39 participants (9 men, 30 women; *M*_age_ = 18.97 years, *SD* = 0.84) in the HP group and 34 participants (12 men, 22 women; *M*_age_ = 19.32 years, *SD* = 1.17) in the LP group.

### Materials

#### Questionnaires

##### Chinese version of the frost multidimensional perfectionism scale

The Chinese version of the frost multidimensional perfectionism scale (CFMPS) was developed by Frost et al. (1990) and translated by Cheng et al. (1999) into its Chinese version. The scale was later revised by Fei and Zhou (2006). The scale contains 27 items and is composed of five subscales, including Concern for Mistakes (CM), Parental Expectations (PE), Doubts About Action (DA), Personal Standards (PS), and Organization (OR). CM, DA, PS, and PE constitute the negative dimensions of the CFMPS (Parker, 1997; Fei and Zhou, 2006), with a higher total score indicating a high level of perfectionism. The five subscales of the CFMPS were shown to have satisfactory internal consistencies (Cronbach α = 0.64–0.76), and acceptable test–retest reliability (0.75–0.82) (Fei and Zhou, 2006). In the current study, the four negative dimensions were found to have adequate internal consistency, with respective alpha coefficients of 0.67, 0.75, 0.76, and 0.88 for CM, DA, PS, and PE. Our criteria for classifying individuals as HP or LP were guided by a previous study reported by Fei et al. (2011), in which the total score of the four negative dimensions was calculated.

##### Chinese version of the depression, anxiety, and stress scale-21 (CDASS-21)

The DASS was developed by Lovibond and Lovibond (1996) and translated into Chinese by Gong et al. (2010). This 21-item scale was administered to determine whether differences in anxiety, depression, or stress exist between HP and LP. All these factors are potential confounding factors, given that attentional response to information is affected by anxiety and depression (Howell et al., 2016).

#### The Stimuli

The experimental picture pairs are taken from Ren and Liu’s (2013) research, including articles of daily use (i.e., mirror, towel, etc.) food, and animals. The perfect pictures were clean, complete pictures downloaded from the Internet. Imperfect pictures were created with Photoshop software to introduce defects with regard to the integrity of the object, the balance of the shape, and the cleanliness of the picture. All image pairs were matched as closely as possible with regard to color, complexity, and brightness. The picture size was standardized to a uniform size (198 × 198 pixels; visual angles were 7.3° × 5.5°), and the centers of the two pictures were separated by 558 pixels, subtending a visual angle of 19.9°.

For each trial in the task, two pictures were presented simultaneously. The picture pair consisted of either (1) an imperfect picture (IP) and a perfect picture (PP) (IP–PP pair) or (2) two perfect pictures (PP–PP pair). Each picture pair was presented only once during the task. PP–PP picture pairs acted as filler to mask the experimental intent, and the relevant data were excluded in statistical analyses. These pictures were assessed on their degree of perfectionism by an independent sample (11 women, 9 men) who did not participate in the eye movement (EM) experiment and were required to respond on a seven-point Likert scale from 1 (very imperfect) to 7 (very perfect). The *t*-test revealed that there was a significant difference between the two types of pictures in their degree of perfection (*t* = 22.516, *p* < 0.001, *M*_pp_ = 5.34, *SD* = 0.54; *M*_IP_ = 3.11, *SD* = 0.54). The final set of pictures consisted of 60 IP–PP pairs and 40 PP–PP pairs. All the images in the experiment were shown only once to avoid the familiarity effect. Presentation of picture pairs was counterbalanced, controlling for the location of PP and IP (left, right) and the presentation order of trials was randomly generated for each participant.

### Apparatus

Participants’ EMs were recorded using an Eye link 1000 EM tracking system (SR Research, Mississauga, ON, Canada). The data were recorded with a sample rate of 1000 Hz. Stimuli were presented on a 17-inch, 65-Hz, 1024 × 768 pixel CRT monitor. Gaze data were acquired from the right eye. A nine-point calibration and validation procedure was conducted prior to starting the task in order to configure the system such that the spatial accuracy error was below 0.5 on average. In addition, calibration accuracy was re-checked after every trial.

### Experimental Procedures

After arriving at the lab and providing informed consent, participants filled out a paper survey consisting of the two questionnaires described earlier. Completion of the questionnaires took from 5 to 10 min. Following completion of the questionnaire, the visual task was introduced and described. To conceal the purpose of the experiment, participants were told that their pupil dilations would be measured (Kellough et al., 2008). The instructions emphasized the claim that participants were taking part in a spatial cognition experiment and that they should look at the “+” that marked the beginning of each trial and to view the images naturally on the screen as if they were watching TV or reading a magazine. Next, prior to the visual task, the participant’s head was kept stable using an adjustable head mount, with a distance between the participant’s eyes and the camera of approximately 65 cm. The eye tracker was then calibrated. Participants first completed six practice trials to adjust to the task followed by the 100 experimental trials. The whole task took approximately 25 min. Upon completion of the task, participants were thanked and were paid 20 Yuan (approximately $3 USD) as compensation for their time.

The protocol for each trial consisted of two stages. At the start of each trial, a black central fixation cross (“+”) appeared at the center of the screen for random time intervals ranging from 600 to 800 ms. Then, a picture pair (IP–PP or PP–PP) was presented. Both pictures in the pair were presented simultaneously, one on the left and the other on the right side of the screen for 2000 ms. The time interval between two adjacent trials was 1000 ms.

### Data Preparation

Prior work has used a 40% criterion for participant exclusion due to excessive signal loss (Graham et al., 2011). In the current study, no participants met this criterion for exclusion. Previous studies (Caseras et al., 2007) also indicated that EM data must meet the condition that saccades be classified as fixations to a position only if they remained stable within a 1° visual angle for at least 100 ms. Fixations on stimuli were identified as effective when the following steps were satisfied: during the initial “+” presentation, participants fixated at the central region; after the presentation of the stimuli pairs, saccades occurred for at least 100 ms; during presentation of the picture pairs, participants fixated on at least one of the pictures instead of other locations around the screen. According to the above criteria, 8.12% trials were excluded from the final analysis. Finally, fixations on either picture during the 2000-ms free-viewing period with durations ≥100 ms were extracted and used for computation of the visual attention indices (Macatee et al., 2017).

### Statistical Analysis

Six visual attention indices were used to assess attentional bias for imperfect pictures and its relation to perfectionism. Two indices measured the initial threat vigilance: orientation of first fixation (the percentage of the first fixation on IP or PP) and latency of first fixation (the time between a picture’s presentation and the making of a fixation). One index represents attentional maintenance: dwell time for each image as the cumulative duration of visual fixations (Shechner et al., 2017). In order to decompose the temporal dynamics of attention allocation across the duration of the prolonged stimulus, each trial of 2000 ms was subdivided into three attentional phases: an early phase (0–500 ms), a middle phase (500–1000 ms), and a late phase (1000–2000 ms), and total fixation duration in each of these three time intervals was calculated.

Independent sample *t*-tests were used to examine whether there were significant differences in age, total score for the negative dimensions of the CFMPS, and scores for anxiety, depression, and stress. To test our hypotheses regarding attentional biases, direction of first fixation on the target image, first fixation latency, and the dwell time and total fixation duration in each of the three attentional phases were analyzed with a 2 × 2 mixed-design repeated-measures analyses of variance (ANOVAs) using group (HP and LP) as the independent samples factor and picture type (perfect and imperfect) as the within-subjects factor.

## Results

### Group Characteristics

The *t*-test showed that there were no significant differences in scores for anxiety and stress between the HP and LP groups [*t*(71) = 1.11, 1.36, both *p* > 0.05, respectively; Table 1]. The difference in depression scores between the two groups was marginally significant [*t*(71) = 1.72, *p* = 0.09], with greater depression scores exhibited in the HP group than in the LP group (Table 1). The HP group had significantly higher values than the LP group for the total score of the negative dimensions of the CFMPS [*t*(71) = 24.02, *p* < 0.001; Table 1]. There was no significant difference in age between the two groups of participants [*t*(71) = −1.44, *p* > 0.05].

**TABLE 1:** Demographic comparisons for participants in HP and LP groups (*n* = 39 vs 34) (means, with standard deviations in parentheses).

	HP group (*n* = 39)	LP	*t*-value	*df*	*p*
Age (years)	18.97 (0.84)	19.32 (1.17)	−1.44	71	0.16
Negative dimensions of CFMPS CCFCFMPS	78.13 (7.98)	35.71 (6.97)	24.02	71	<0.001
DASS-21 (depression)	2.33 (1.30)	1.85 (1.05)	1.72	71	0.09
DASS-21 (anxiety)	2.79 (1.44)	2.44 (1.26)	1.11	71	0.27
DASS-21 (stress)	4.69 (1.61)	4.15 (1.83)	1.36	71	0.18

### EM Results

#### Initial Visual Attention Orientation

**FIGURE 1:**
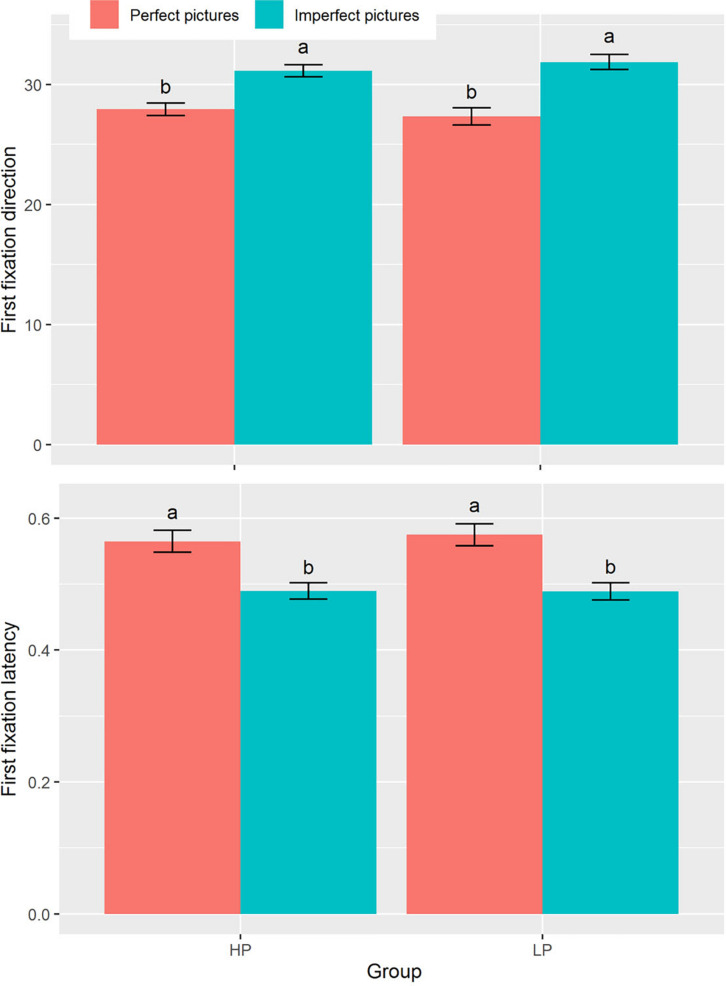
First fixation direction **(upper panel)** and first fixation latency **(bottom panel)** under combinations of group (HP vs LP) and picture types (perfect pictures vs imperfect pictures). The different lowercases denote significant difference at *p* < 0.05. Data are presented as mean ± SE.

The 2 × 2 mixed-design repeated-measures ANOVA showed that the main effect of picture type significantly impacted first fixation direction [*F*(1, 71) = 23.56, *p* < 0.001, η_p_^2^ = 0.25, Table 2]. There was a greater tendency for all participants to orient initially toward imperfect pictures rather than perfect pictures (*M* ± *SD* = 31.49 ± 3.36 and 27.67 ± 3.67, respectively). Similarly, the main effect of picture type significantly affected first fixation latency [*F*(1,71) = 122.50, *p* < 0.001, η_p_^2^ = 0.64, Table 2]: both the HP and LP groups had shorter (i.e., faster) initial fixation latencies on imperfect pictures than on perfect pictures (*M* ± *SD* = 0.49 ± 0.08 and 0.57 ± 0.10, respectively). However, neither the interaction between picture type and group nor group itself significantly affected the first fixation direction and first fixation latency (see Figure 1).

**TABLE 2:** Summary of eye-movement data.

EM Index	*F* (η_p_^2^)		
	
	Group	Picture type	Picture type × group
**Initial visual attention orientation**			
First fixation direction (N)	0.09 (0.001)	23.56***(0.25)	0.69 (0.01)
First fixation latency(S)	0.059 (0.001)	122.50***(0.64)	0.52 (0.007)
Maintenance of visual attention			
Total dwell time(S)	0.21 (0.003)	57.62***(0.45)	11.18***(0.14)
**Attentional phases**			
Early phase (0–500 ms) (S)	1.15 (0.02)	39.97***(0.36)	0.25 (0.004)
Middle phase (500–1000 ms) (S)	0.02 (0.000)	134.20***(0.65)	1.84 (0.03)
Late phase (1000–2000 ms) (S)	1.07 (0.02)	18.09***(0.20)	11.76***(0.14)

*p < 0.05, **p < 0.01, ***p < 0.001.

#### Maintenance of Visual Attention

**FIGURE 2:**
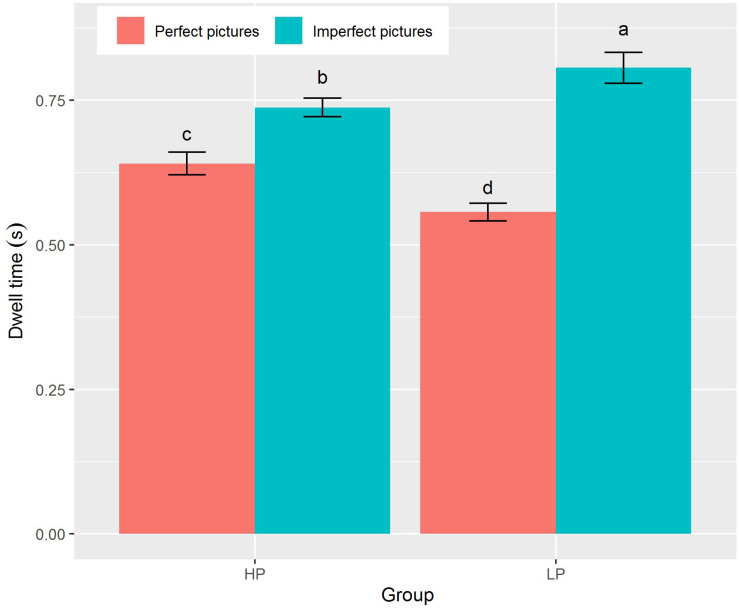
Dwell time under combinations of group (HP vs LP) and picture types (perfect pictures vs imperfect pictures). The different lowercases denote significant difference at *p* < 0.05. Data are presented as mean ± SE.

The main effect of picture type was significant for total dwell time [*F*(1,71) = 57.62, *p* < 0.001, η_p_^2^ = 0.45, Table 2]: both the HP and the LP groups exhibited longer dwell time for imperfect pictures than for perfect pictures (*M* ± *SD* = 0.77 ± 0.13 and 0.60 ± 0.12, respectively). The effect of the interaction between group and picture type on dwell time was significant [*F*(1,71) = 11.18, *p* = 0.001, η_p_^2^ = 0.14]. The LP group showed longer dwell time toward imperfect pictures than did the HP group (*M* ± *SD* = 0.81 ± 0.16 and 0.74 ± 0.10, respectively), while the HP group showed longer dwell time than the LP group toward perfect pictures (*M* ± *SD* = 0.64 ± 0.12 and 0.56 ± 0.09, respectively). There was no significant main effect of group on dwell time (see Figure 2).

### Attentional Phases

**FIGURE 3:**
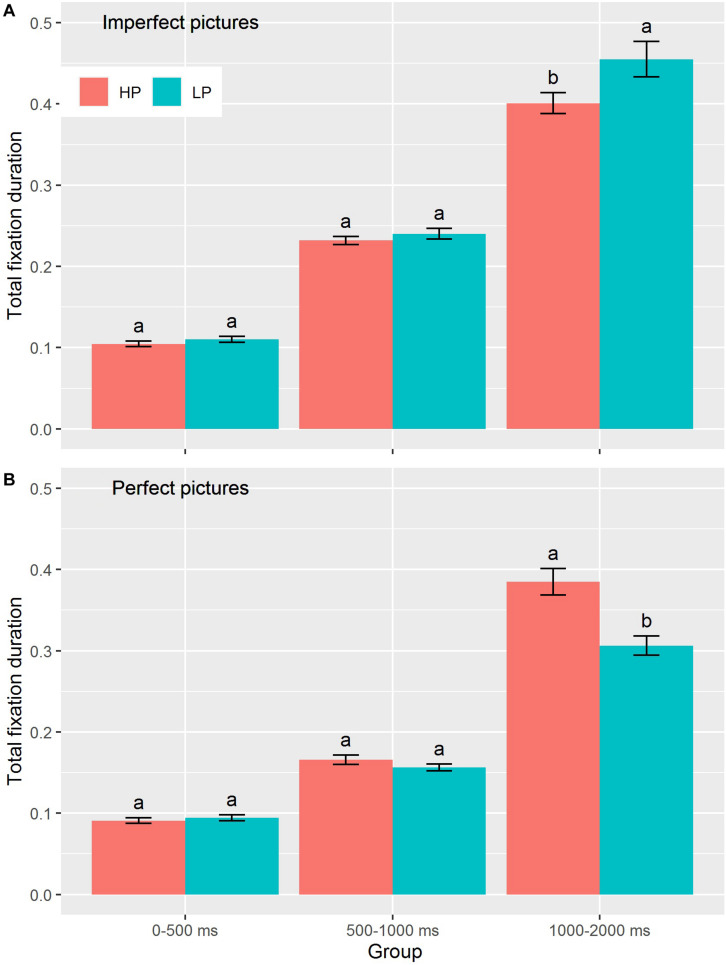
Total fixation duration between HP and LP groups across three consecutive time intervals toward imperfect pictures **(A)** and perfect pictures **(B)**. The different lowercases denote significant difference for each phases of three consecutive time intervals at *p* < 0.05. Data are presented as mean ± SE.

For early-phase total fixation duration (0–500 ms), the main effect of picture type was significant [*F*(1,71) = 39.97, *p* < 0.001, η_p_^2^ = 0.36, Table 2]. All participants had longer total fixation duration for imperfect pictures than for perfect pictures (*M* ± *SD* = 0.11 ± 0.02 and 0.09 ± 0.02, respectively) in the early phase. Similarly, for middle-phase total fixation duration (500–1000 ms), the main effect of picture type was also significant [*F*(1,71) = 134.20, *p* < 0.001, η_p_^2^ = 0.65]: all participants had longer total fixation duration for imperfect pictures than for perfect pictures (*M* ± *SD* = 0.24 ± 0.04 and 0.16 ± 0.03, respectively). However, neither the main effect of group nor the interaction between group and picture type significantly affected total fixation duration in the early and middle phases. For late-phase total fixation duration (1000–2000 ms), the main effect of picture type was significant [*F*(1,71) = 18.09, *p* < 0.001, η_p_^2^ = 0.20, Table 2] in that the total fixation duration for imperfect pictures was significantly longer than that for perfect pictures (*M* ± *SD* = 0.43 ± 0.11 and 0.35 ± 0.10, respectively). The interaction between picture and group also had a significant effect on late-phase total fixation duration [*F*(1,71) = 11.76, *p* = 0.001, η_p_^2^ = 0.14, Table 2]. Simple main effects showed that the total fixation duration for imperfect pictures was significantly longer for the LP group than for the HP group in the late phase (*M* ± *SD* = 0.45 ± 0.13 and 0.40 ± 0.08, respectively). Conversely, the total fixation duration for perfect pictures was significantly longer for the HP group than for the LP group in the late phase (*M* ± *SD* = 0.39 ± 0.10 and 0.31 ± 0.07, respectively). There was no significant main effect of group on late-phase fixation duration (see Figure 3).

## Discussion

To the best of our knowledge, this is the first experimental study to use EM measurements to investigate the attentional patterns associated with perfectionism during exposure to perfect–imperfect picture pairs. The results showed that both the HP and the LP group had an overall attentional bias toward imperfect pictures based on their initial visual attention orientation and their attentional maintenance. A further examination of the results from each of the three phases of attentional processing showed that there was no significant difference between the HP group and the LP group during the first 1000 ms of stimulus presentation, during which time both groups paid more attention to the imperfect pictures. However, during the second 1000 ms, the participants in the HP group diverted their attention away from the imperfect pictures and began to pay more attention to the perfect pictures.

The initial vigilance toward the imperfect pictures by all participants could be attributed to the following reasons. First, perfect things are more in line with people’s expectations. Adler (1956) once claimed that “striving for perfection is innate in the sense that it is a part of life, a striving, an urge, a something without which life would be unthinkable, this feeling of longing for the abrogations of every imperfection, is never absent”. Thus, both groups showed an initial vigilance to the imperfect pictures. Second, imperfect stimuli are visually more complex, and detecting flaws in imperfect pictures may consume more attentional resources. This would result in all participants having longer total fixation durations for imperfect pictures than for perfect pictures in the early and middle attentional phases. Our findings of attentional bias toward imperfect stimuli during the HP group’s early exposure are consistent with previous behavioral studies (Fei et al., 2011; Ren and Liu, 2013; Howell et al., 2016). However, the LP group presented the same pattern as the HP group in the early phase; this result was opposite to earlier studies that showed that LP groups display attentional avoidance to imperfect stimuli (Fei et al., 2011; Ren and Liu, 2013). One possible explanation is that the response latency tasks that were used in past studies to assess attentional bias only indirectly captured a single “snapshot” of attention at a time point of 500 or 750 ms. The measurements may thus have been confounded by motor retardation. Meanwhile, the different components of attention (e.g., orienting attention vs maintenance of attention) could not be differentiated in response latency tasks (Mathews et al., 1996).

Both groups showed attention maintenance to imperfect pictures, but a significant difference between the HP and the LP groups developed as the stimulus presentation progressed over time. During the late attentional phase, the participants in the HP group diverted their attention away from the imperfect pictures and began to pay more attention to the perfect pictures. This differential pattern of attention over time supports the conclusions drawn from Crombez et al. (2013) that “conscious and elaborative processes are critical for attentional biases to emerge.” Stimuli were displayed for a relatively long time (2000 ms) in our study. These passive viewing conditions may have engaged emotional regulation strategies in the late phase (Shechner et al., 2017). Previous studies have found that perfectionists experience imperfection anxiety when they process imperfect stimuli, e.g., damaged, unfinished, or untidy stimuli (Fei et al., 2011; Ren and Liu, 2013). Imperfect pictures might, therefore, evoke imperfection anxiety in HP participants, causing voluntary strategic avoidance. Previous studies have shown that self-critical perfectionists were more likely to choose avoidant strategies to distract attention from threatening stimuli (Dunkley et al., 2003). Visual avoidance may be considered to be a strategic, top-down process by which individuals in the HP group attempt to minimize the aversive impact triggered by imperfect stimuli. However, because anxiety was not measured in response to the images, this interpretation is still speculative and should be examined in future studies.

A detailed understanding of the attentional bias of perfectionism is key to clinical prevention and intervention. The present study has important clinical implications regarding the treatment of high perfectionism. The results showed that HP individuals tend to avoid imperfect stimuli in the later phase of attention, indicating that avoidance might play an important role in maintaining perfectionism. Although attention avoidance in later stages of processing was the result of emotional management, these avoidant behaviors do not result in a complete resolution of anxiety and conversely may lead to enduring symptoms (Koster et al., 2005). Interventions for HP should, therefore, focus on developing alternative opinions about imperfection. Perhaps once HP individuals are better able to accept imperfect stimuli, the general symptoms caused by perfectionism such as distress, anxiety, and depression will be reduced.

We also acknowledge that there are several limitations to the present study. First, individual differences in perfectionism were assessed using the CFMPS. Although the CFMPS consistently has very good reliability and validity, it cannot necessarily screen out implicit perfectionists who pursue perfectionism unconsciously. Future research should incorporate the interview or other methods to measure perfectionism and to examine attentional bias in individuals drawn from clinical samples. Second, the numbers of IP and PP were not exactly matched because some perfect pictures were used as fillers. This may have introduced a frequency effect that would cause participants to look at the imperfect pictures more often. Third, the sample in our study was only a non-clinical sample, and this could limit the generalizability of our findings. Future studies should examine the attentional bias in clinical samples. Fourth, only HP and LP were compared in this study. Based on the 2 × 2 model of perfectionism (Gaudreau and Thompson, 2010), four subtypes of perfectionism could be differentiated. Future research should recruit participants from across the full distribution of perfectionism scores and examine the differences in attentional biases among the four subtypes.

## Conclusion

It can be concluded that the participants in the HP group tended to avoid imperfect stimuli in the later phase of attention, indicating that avoidance might play an important role in maintaining perfectionism. We hope that there will be more studies on perfectionist cognitive biases in the future by means of eye-tracking technology across various groups with different degrees of attentional bias. In addition, we conclude that the application of eye-tracking technology can be an aid to monitoring the progress and efficacy of clinical interventions.

## Data Availability Statement

All datasets presented in this study are included in the article/Supplementary Material.

## Ethics Statement

The studies involving human participants were reviewed and approved by Ethics Committee in Faculty of Psychology, Tianjin Normal University. The patients/participants provided their written informed consent to participate in this study.

## Author Contributions

JL and XPL designed the study, performed the measurement, and drafted the manuscript together. XCL conducted literature searches. WT was responsible for the interpretation of the data. BY revised the language. All authors contributed to the article and approved the submitted version.

## Conflict of Interest

The authors declare that the research was conducted in the absence of any commercial or financial relationships that could be construed as a potential conflict of interest.
