# Supplementary material for: Attentional Bias for Imperfect Pictures in Perfectionism: An Eye-Movement Study
Source: Front Psychol. 2020 Oct 2;11:566482. doi: 10.3389/fpsyg.2020.566482 (PMC7561667; doi:10.3389/fpsyg.2020.566482)
Supplement: Supplementary file 1 [file Table_1.DOCX]

**Table 1** Demographic comparisons for participants in HP and LP groups (n = 39 versus 34) (means, with standard deviations in parentheses).

|  | HP Group(n=39) | LP | *t* value | | *df* | *p* |
| --- | --- | --- | --- | --- | --- | --- |
| Age（years） | 18.97 (0.84) | 19.32 (1.17) | | -1.44 | 71 | 0.16 |
| Negative dimensions of CFMPS CCFCFMPS | 78.13 (7.98) | 35.71 (6.97) | | 24.02 | 71 | <0.001 |
| DASS-21 (depression) | 2.33 (1.30) | 1.85 (1.05) | | 1.72 | 71 | 0.09 |
| DASS-21 (anxiety) | 2.79 (1.44) | 2.44 (1.26) | | 1.11 | 71 | 0.27 |
| DASS-21 (stress) | 4.69 (1.61) | 4.15 (1.83) | | 1.36 | 71 | 0.18 |

**Table 2** Summary of eye-movement data

| EM Index | F（η_p_^2^） | | |
| --- | --- | --- | --- |
|  | Group | Picture type | Picture type×Group |
| Initial visual attention orientation |  |  |  |
| First fixation direction (N) | 0.09 (0.001) | 23.56^**^^*^ (0.25) | 0.69 (0.01) |
| First fixation latency(S) | 0.059 (0.001) | 122.50^***^ (0.64) | 0.52 (0.007) |
| Maintenance of visual attention |  |  |  |
| Total dwell time(S) | 0.21 (0.003) | 57.62^***^ (0.45) | 11.18^***^ (0.14) |
| Attentional phases |  |  |  |
| Early phase (0–500ms) (S) | 1.15 (0.02) | 39.97^***^ (0.36) | 0.25 (0.004) |
| Middle phase (500–1,000ms) (S) | 0.02 (0.000) | 134.20^***^ (0.65) | 1.84 (0.03) |
| Late phase (1,000–2,000ms) (S) | 1.07 (0.02) | 18.09^***^ (0.20) | 11.76^***^ (0.14) |

^*^p < 0.05, ^**^p < 0.01, ^***^p < 0.001
